# 

**Published:** 1912-09

**Authors:** 


*THE BRISTOL MEDICO-CHIRURGICAL SOCIETY.*

*Editor:* R. SHINGLETON SMITH, M.D., B.Sc.
WITH WHOM ARE ASSOCIATED
J. MICHELL CLARKE, M.A., M.D.,
J. LACY FIRTH, M.S., M.D., JAMES SWAIN, M.S., M.D.
P. WATSON-WILLIAMS, M.D., *Assistant Editor.*
*Editorial Secretary:* J. A. NIXON, B.A., M.B.

LIBRARY OF CONGRESS
SEP 30 1912
SMITHSONIAN DEPOSIT.

### CONTENTS.

*Page*

Tuberculosis. By ROBERT ROXBURGH, M.B. Edin. ... ... ... 193

Some Remarks on Insanity; its Classification and Nomenclature. By F. St. JOHN BULLEN, M.R.C.S. Eng. ... ... ... ... 205

The Right and the Wrong Side of the X-Ray Picture; Has a Mistake been Made? By W. COTTON, M.A., M.D. ... ... 227

The Relationship between Toxicity and Chemical Reactivity in Certain Benzene Derivatives. By OLIVER C. M. DAVIS, D.Sc. ... 237

Raynaud's Disease. By DAVID A. ALEXANDER, M.B., Ch.B. Vict. ... 247

Progress of the Medical Sciences:

Medicine. By J. A. NIXON, B.A., M.B., B.C. Cantab. ... ... 251

Surgery. T. CARWARDINE, M.B., M.S. Lond. ... ... ... ... 257

Ophthalmology. A. OGILVY, M.D., B.Ch., B.A. Dub. ... ... 259

Reviews of Books ... ... ... ... ... ... ... ... 262

Editorial Note ... ... ... ... ... ... ... ... ... 274

Notes on Preparations for the Sick ... ... ... ... ... 276

The Library of the Bristol Medico-Chirurgical Society ... ... 280

Meetings of Societies ... ... ... ... ... ... ... ... 282

Obituary ... ... ... ... ... ... ... ... ... ... 285

Local Medical Notes ... ... ... ... ... ... ... ... 287

